# Beyond Individual Coping: The Role of Social Capital in Community-Based Mental Health Support for Displaced Somali Youth

**DOI:** 10.3390/ijerph22050784

**Published:** 2025-05-15

**Authors:** Hyojin Im, Shinhye Lee, Abdulkadir Warsame, Maimuna Isse

**Affiliations:** 1 School of Social Work, Virginia Commonwealth University, Richmond, VA 23284, USA; 2 Department of Social Welfare, Seoul National University, Seoul 08826, Republic of Korea; selfpitysh@gmail.com; 3 Tawakal Medical Centre, Nairobi 51529-00200, Kenya

**Keywords:** urban refugees, Somali youth, social capital, help-seeking, social support, stress and coping, mental health and psychosocial support

## Abstract

Somali refugee youth face intersecting stressors related to displacement, economic hardship, and systemic exclusion, yet their coping strategies remain understudied. This study examines the psychosocial distress, coping mechanisms, and help-seeking behaviors of Somali refugee youth in Nairobi’s urban displacement context using a mixed-methods approach. Quantitative analyses assess the prevalence of stressors, coping strategies, and perceived support, while qualitative responses provide insight into lived experiences. Findings reveal that 72% of participants reported distress linked to economic insecurity (15.9%), family separation (16.9%), or refugee-related adversities (13.2%). Religious coping (59.5%) and self-care practices (60.5%) were the most frequently used strategies, while 15.8% relied on avoidance-based methods. Help-seeking patterns showed that 76.7% of participants were willing to seek help in general, but only 40.7% did so for emotional issues, with mothers and same-sex friends being primary sources. Regression analysis (R^2^ = 0.507, *p* < 0.001) showed that scope of community (β = 0.417, *p* = 0.001), trust in bonding social capital (β = 0.343, *p* = 0.012), and perceived community violence (β = 0.346, *p* = 0.003) were positively associated with perceived support. In contrast, help-seeking intention (β = −0.206, *p* = 0.049) was negatively associated with perceived support. Other variables—including religious coping, emotional coping, age, gender, and bridging trust—were not significant predictors. These findings underscore the need for community-driven mental health interventions that strengthen existing social structures while integrating culturally adapted service models. Leveraging social capital is essential for fostering sustainable, accessible, and community-based mental health support for displaced Somali youth.

## 1. Introduction

Refugee youth constitute one of the most vulnerable subgroups within displaced populations due to the cumulative and intersecting effects of developmental transitions, pre- and post-migration adversities, and systemic inequities in host societies [1]. Their stressors are multi-layered, encompassing exposure to violence and trauma in their countries of origin, precarious migration journeys, and structural and social exclusion in resettlement contexts [2,3,4]. These adversities heighten their risk for psychopathology, with elevated rates of depression, anxiety, and post-traumatic stress disorder (PTSD) observed in displaced youth [5]. These adversities heighten refugee youths’ risk for mental health challenges, which, as defined by the World Health Organization [6], include not only the absence of illness but also the presence of psychological well-being—the ability to regulate emotions, form relationships, and manage stress. In displacement contexts, mental health symptoms are often shaped by structural adversity and cultural norms, making distress less visible through Western diagnostic lenses [7]. Emotional distress, characterized by internalized worry, sadness, and tension, is frequently somatized or unspoken in collectivist societies due to stigma and expectations of emotional restraint [7,8]. However, refugee youth are not passive recipients of distress; rather, they actively deploy diverse coping strategies shaped by individual, cultural, and environmental factors, influencing their psychosocial trajectories [9,10,11,12].

Coping, broadly defined, refers to the cognitive and behavioral efforts individuals use to manage internal and external demands that are perceived as stressful or overwhelming [13]. Lazarus and Folkman’s seminal model distinguishes between problem-focused coping (addressing the source of stress), emotion-focused coping (regulating emotional responses), and avoidance coping (disengaging from the stressor) [14]. Among refugee youth, coping strategies are shaped by structural constraints, cultural norms, and collective expectations [15]. Informal support systems, such as family, peers, and broader community networks, are central to emotional regulation and resilience, particularly in displacement settings where professional mental health care is limited or culturally misaligned [16,17]. Among many refugee youth, religious coping mechanisms further mediate experiences of distress, offering spiritual meaning-making and structured guidance [18,19,20]. Faith-based practices, such as prayer, Quran recitation, and participation in religious community spaces, are deeply ingrained in many displaced populations, serving as vital sources of resilience and collective coping [21,22].

Despite the centrality of informal and religious coping strategies, the underutilization of formal mental health services remains a pressing concern. Structural and cultural barriers, including mental health stigma, linguistic challenges, cultural incongruence, and institutional mistrust, often inhibit refugee youth from seeking professional psychosocial support [23,24]. Additionally, many youth prioritize self-reliance or avoidance-based strategies, ranging from relatively neutral approaches such as cognitive distraction and emotional suppression to more harmful behaviors such as self-medication [25,26]. While these strategies may offer short-term relief, they often contribute to long-term psychological burden and hinder access to sustainable mental health support.

Beyond individual coping mechanisms, social capital provides a theoretical lens for understanding how support networks function within refugee communities [27,28]. Social capital refers to the resources embedded within social relationships that can be mobilized to access emotional, informational, or practical support. It includes bonding social capital (close, trusting relationships within one’s community) and bridging social capital (connections across different ethnic, religious, or institutional groups) [29]. Both forms play distinct roles in shaping access to care and resilience under adversity [30]. Among displaced youth, the quality of social relationships, trust in community members, and active engagement in communal life significantly shape their access to material and emotional support [10,31,32]. The distinction between bonding social capital, which consists of close-knit networks within ethnic or religious communities, and bridging social capital, which includes relationships extending beyond one’s cultural or ethnic in-group, is particularly relevant in understanding perceived support and well-being outcomes [29]. Strong in-group ties provide immediate emotional security, but excessive insularity may limit broader social integration, while bridging social capital can enhance economic and social mobility, albeit with challenges in trust-building and accessibility [33].

### Stress and Coping Among Somali Refugee Youth

As one of the largest protracted refugee populations globally, Somali refugees have endured decades of forced displacement due to conflict, economic instability, and environmental crises in the Horn of Africa [34]. Somali refugee youth, often separated from extended kinship structures, must navigate the compounded stressors of familial loss, disrupted education, and economic hardship in host countries, with these challenges further intensified by prolonged displacement, fragmentation of family, and community cohesion [35,36]. In urban resettlement contexts, for example, these challenges are compounded by economic precarity, structural racism, and the erosion of traditional social networks, which further intensify psychosocial distress and social exclusion [37,38]. In the Somali community, religious coping remains a defining feature of stress management, extending beyond personal spirituality to reinforce a collective sense of resilience through faith [39,40]. Perceived as a first-line intervention, religious coping is often prioritized over secular mental health services, which, while fostering psychological strength, do not fully substitute for social capital, particularly in environments where systemic inequities pose persistent challenges [39]. Without robust social networks, reliance on religious coping alone may leave youth vulnerable to isolation, limiting their access to practical resources and broader support systems necessary for long-term well-being [41].

Despite the centrality of religious and familial coping, Somali refugee youth face persistent barriers to accessing formal mental health care. Mental health stigma, limited awareness of available services, and concerns over confidentiality contribute to widespread reluctance to seek professional support [42,43]. Additionally, institutional mistrust—shaped by experiences of systemic discrimination and inadequate service provision—further reinforces a preference for informal networks [44,45]. While familial and community-based support structures provide essential emotional and social resources, they may not always be equipped to address more complex psychosocial challenges, particularly in urban displacement contexts where traditional networks are disrupted [7].

Given these dynamics, understanding the interplay between stress, coping, and perceived support is essential for informing culturally responsive mental health interventions. While prior studies have explored refugee mental health broadly, there remains a lack of research that explicitly connects emotional distress, coping behavior, and perceived support to different forms of social capital in displaced urban youth populations. This study addresses this gap by investigating how Somali refugee youth interpret, experience, and respond to psychosocial distress, and how these processes are embedded within structural and relational contexts. Using a mixed-methods approach, this study examines the stressors, coping mechanisms, and help-seeking behaviors of Somali refugee youth in Nairobi’s urban displacement setting. The quantitative component assesses the prevalence of key psychosocial stressors, coping strategies, and perceived support, while the qualitative analysis provides deeper insight into the lived experiences shaping these patterns. Specifically, this study aims to (1) identify primary sources of emotional distress, (2) explore coping strategies, including religious, social, and avoidance-based mechanisms, (3) examine help-seeking behaviors and barriers to formal support, and (4) analyze predictors of perceived support, with a particular focus on bonding and bridging social capital. By integrating these findings, the study contributes to a more nuanced understanding of resilience and vulnerability among Somali refugee youth and offers critical insights for developing culturally grounded mental health interventions in urban displacement settings.

## 2. Methods

According to UNHCR [46], Somali refugees represent 280,156 of the 529,854 registered refugees and asylum seekers in Kenya, with approximately 83,000 residing in Nairobi’s urban settings, predominantly in Eastleigh, an area often referred to as “Little Mogadishu” [35,47,48]. As the largest Somali community outside Somalia, Eastleigh has served as a sanctuary for Somali refugees seeking economic stability, educational opportunities, and social support, yet the area remains characterized by pervasive insecurity, discrimination, and systemic exclusion from formal services [35,49]. This project was implemented in collaboration with a community-based organization (CBO) in Eastleigh, led by Somali healthcare professionals, counselors, and community leaders who played an integral role in the research process. The involvement of local stakeholders ensured that the study’s objectives, methodology, and implementation were culturally appropriate, ethically sound, and responsive to the realities of the target population.

### 2.1. Participants and Procedure

Participants were recruited using snowball sampling, a method chosen due to the highly mobile and often insecure status of urban refugees. Inclusion criteria required participants to be Somali refugees or asylum seekers residing in Eastleigh and between the ages of 15 and 25. A semi-structured survey questionnaire was administered by trained Somali counselors and youth community leaders (three female and two male facilitators). Participants could complete the survey independently or opt for oral administration, with responses electronically recorded by facilitators when necessary. To ensure accessibility, the survey was available in both Somali and English, allowing participants to choose their preferred language. A total of 189 Somali refugee youth participated in the study, of whom 55 (29.1%) identified as male, 81 (42.9%) as female, and 53 (28.0%) did not report their gender. This study was reviewed and approved by both the Institutional Review Board at the first author’s institution and a Community Advisory Board composed of Somali refugee leaders and local service providers based in Eastleigh. Given the precarious legal and social status of many Somali refugees in Nairobi, the research team emphasized confidentiality and assured participants that their responses would remain anonymous and would not be shared with authorities or external entities.

### 2.2. Measures

The semi-structured survey comprises two primary sections: (a) stress and coping strategies and (b) social capital and perceived social support. The survey was developed in collaboration with Somali community stakeholders, including counselors, youth leaders, and healthcare professionals, to ensure cultural and contextual relevance. The open-ended items were co-developed through a participatory process involving brainstorming sessions with community leaders and pre-tested with Somali youth for clarity and cultural resonance before finalization. The instrument was available in both English and Somali, with translation and back-translation conducted by bilingual professionals.

#### 2.2.1. Stress and Coping Strategies

To assess emotional distress, participants responded to an open-ended prompt: “What makes you most worried these days?” This qualitative approach allowed for the identification of distress sources, including economic insecurity, family-related stressors, and community violence. Coping strategies were explored through the question: “What do you think is helpful to reduce your worries, distress, and emotional distress or suffering? List three things that you usually do.” Help-seeking behaviors were also assessed using a series of questions examining participants’ willingness to seek assistance: “Are you willing to ask for help from others or share your emotional suffering?” (Yes/No). Participants were additionally asked to specify their preferred sources of support, including family, friends, religious leaders, professionals, or community members. Because these were open-ended items, traditional measures of internal consistency (e.g., Cronbach’s alpha) were not applicable. Instead, rigor was ensured through structured coding procedures, coder consensus, and community validation, as detailed in the Section 2.3.

#### 2.2.2. Social Capital and Perceived Social Support

Social capital was assessed through participants’ perceptions of their community and trust in social networks. Participants first answered an open-ended question: “What does community mean to you?” and then selected from predefined categories identifying their community members. These categories included immediate family, extended relatives, friends, members of the same Somali clan, members of other Somali clans, Somali people in Eastleigh, Kenyan locals, and broader diaspora groups. Trust within social networks was categorized into bonding social capital (in-group trust within Somali networks) and bridging social capital (trust in individuals outside the Somali ethnic group). Perceived sources of help were also measured in relation to these social capital constructs, allowing for an analysis of whether strong bonding ties within the Somali community facilitated greater support or whether bridging ties expanded perceived social support.

Perceived social support was measured using the Multidimensional Scale of Perceived Social Support (MSPSS) [50], a widely used instrument assessing support from family, friends, and significant others. Based on feedback from community stakeholders, 10 of the original 12 items were retained to enhance cultural relevance and remove redundancy. Items were rated on a five-point Likert scale (1 = *strongly disagree* to 5 = *strongly agree*), with higher scores indicating greater perceived support. The MSPSS demonstrated strong internal consistency in this sample (Cronbach’s α = 0.867).

### 2.3. Data Analysis

Data analysis followed a mixed-methods approach, integrating thematic analysis for qualitative responses with statistical modeling for quantitative data to capture the complexity of Somali refugee youth’s experiences of distress, coping, and social support. The qualitative data were analyzed using thematic analysis following Braun and Clarke’s six-phase framework [51]. Two authors independently conducted initial coding, iteratively refining categories through discussion to develop a structured codebook. Discrepancies were resolved through consensus, ensuring intercoder reliability [52]. This process, combined with community review and codebook validation, supports the trustworthiness of the qualitative data by enhancing credibility, dependability, and confirmability in line with best practices in qualitative research. To enhance credibility and cultural validity [53], two Somali community researchers reviewed and refined the codes during a field visit to Eastleigh, ensuring alignment with local conceptualizations of stress, coping, and social support. The finalized thematic framework was systematically applied across all qualitative responses, with recurrent themes analyzed in relation to the quantitative findings.

Quantitative analyses were conducted using IBM SPSS Statistics Version 27, beginning with descriptive analyses to summarize demographic characteristics, sources of distress, coping mechanisms, and perceived support. Bivariate analyses, including Pearson’s correlations and independent *t*-tests, assessed relationships among key variables. A multiple regression analysis examined predictors of perceived support, including bonding and bridging social capital, emotional and religious coping, help-seeking behaviors, perceived community violence, and scope of community. Diagnostic checks confirmed assumptions of normality, linearity, and homoscedasticity, with variance inflation factors (VIFs) below 2. Adjusted R^2^ values were reported to assess model fit, and significance was set at *p* < 0.05. The integration of qualitative and quantitative findings allowed for triangulation, enhancing both the interpretive depth and the validity of the study’s conclusions regarding the role of social capital and coping mechanisms in shaping perceived support among Somali refugee youth.

## 3. Results

Table 1 presents the demographic characteristics of the participants and provides an overview of key quantitative measures, offering a foundational context for the subsequent analyses.

### 3.1. Sources of Emotional Distress

The findings reveal a diverse range of distress sources reported by displaced Somali youth, reflecting both individual and structural challenges encountered in their daily lives (see Table 2). While 28.0% (n = 53) reported no significant distress, indicating resilience or the absence of immediate stressors, the majority cited socio-economic, familial, and displacement-related concerns. Basic needs, livelihood, and education challenges (15.9%) were prevalent, with concerns ranging from financial instability to academic pressures, as one participant noted, *“I worry about my fee for my IT classes,”* and another, *“Job, I am worried about job, I am jobless, and it’s the only worry I have.”* Refugee-related adversities (13.2%) reflected persistent fears of violence and systemic insecurity, including *“police harassment and violence happening in my community”* and *“I am scared of the people who once tried to kill my father and harm my family to come back.”* Family-related issues (16.9%) centered on separation and caregiving concerns, as illustrated by one respondent’s distress over *“my sick children”* and another’s worry about *“my family because I am not with them, especially my mum.”* Despite the stigma surrounding mental health and emotional expression, 7.4% explicitly referenced psychological distress, often linked to intrusive thoughts and uncertainty. One participant described *“the life I am in is making me worry so much,”* while another struggled with *“forgetting and thinking a lot.”* Community and Somalia-related concerns (5.3%) reflected transnational anxieties, with some respondents preoccupied with *“the Somali youth who are suffering all over the world”* or *“the conflict between the Somali community.”* A 13.2% missing response rate may indicate reluctance to disclose distress or difficulty articulating experiences.

### 3.2. Coping Strategies

As shown in Table 3, Somali youth predominantly rely on religious coping (59.5%) and self-care practices (60.5%), underscoring the centrality of faith and personal regulation in managing stress. Prayer, Quran recitation, and mosque visits are common coping mechanisms, as several respondents noted: *“I read the Quran to feel better”* and *“I pray toward Mecca and seek guidance.”* Self-care strategies, both physical (30%) and psychological (23.7%), reflect a strong preference for self-regulation. Many youth turn to simple yet essential practices such as sleeping, resting, or drinking water to manage stress, as one participant described: *“I just rest, take a bath, and drink water to calm down.”* Psychological self-care includes self-encouragement, journaling, and self-reflective reading, as expressed in statements such as *“I read books about depression to help myself”* and *“I try to minimize my thoughts by reading my diary.”* A smaller proportion (6.8%) engages in meditation and breathing exercises, indicating a growing awareness of mindfulness practices as a coping mechanism.

Beyond individual strategies, social coping (25.8%) is also common, with most youth turning to friends and family (22.6%) for support. This reliance on interpersonal relationships is reflected in statements like *“I go out with friends to feel better”* and *“I see someone I love to get through my problems.”* However, prosocial behaviors (3.2%), such as helping others, are less frequently mentioned, suggesting that while social engagement provides comfort, few respondents actively seek emotional relief through altruistic acts. Despite the presence of social coping, help-seeking behaviors (56.4%) overwhelmingly favor informal networks (39%) over formal avenues such as counseling, school-based support, or rehabilitation (12.1%). As one participant articulated, *“I prefer talking to someone in my community rather than going to a professional”*, which reflects a reluctance to engage with institutional support systems. A significant proportion of youth (15.8%) also employs avoidance-based strategies, including distraction (8.4%) and emotional suppression (7.4%). These responses are evident in statements such as *“I make myself busy so I don’t think about it”* and *“I try to forget and avoid thinking about the problem.”* Furthermore, emotional outbursts (5.3%), such as crying or screaming, highlight acute distress and a lack of accessible coping mechanisms for some youth: *“I cry alone in a dark room”* and *“I scream when I feel overwhelmed.”* These patterns suggest that interventions must leverage existing faith and community structures while addressing the risks associated with avoidance-based coping and barriers to formal support (see Table 3 for details).

### 3.3. Seeking Help for Emotional Distress: Sources, Motivations, and Barriers to Help-Seeking

Aligned with the reported coping strategies, Somali youth overwhelmingly rely on informal support systems when sharing personal problems, with formal avenues of help-seeking remaining marginal. As shown in Table 4, the most frequently cited confidants are same-sex friends (37.4%) and family members (25.8%), reinforcing the centrality of close-knit, trusted relationships in coping with distress. Among family members, mothers (8.9%) and parents collectively (16.8%) are the most frequently mentioned sources of support, while siblings (2.6%) and spouses (0.5%) are much less common, implying collectivist cultures, where familial bonds are integral to emotional regulation and problem-solving.

Beyond immediate kinship networks, community members (12.6%) also serve as sources of support, though their role appears more diffuse, with some respondents specifying “someone else” without clear identification. Interestingly, religious figures (1.1%) were infrequently mentioned, despite the broader recognition of religious coping in Somali culture. This suggests that, while spirituality remains an important coping mechanism, direct reliance on religious authorities for problem-sharing may be limited.

In contrast, formal sources of support—teachers (4.7%) and professionals (3.2%)—were rarely sought out, underscoring persistent barriers to institutional engagement. Structural, cultural, and systemic factors likely contribute to this pattern, including stigma surrounding professional mental health services, distrust of institutions, and a preference for culturally familiar support systems. These findings highlight the dominance of peer and familial relationships in Somali youth’s help-seeking behaviors, suggesting that interventions aimed at improving psychosocial support must leverage existing informal structures rather than relying solely on formal service provision.

Building on the themes discussed in the preceding section, Table 5 further illustrates the complex and often contradictory dynamics of help-seeking behaviors among Somali youth, revealing how trust, emotional needs, and self-reliance collectively shape their decisions to seek or avoid support. The subsequent subsections delve more deeply into these dynamics by examining (1) perceived availability and efficacy of support, (2) trust, safety, and emotional relief, and (3) the interplay between interpersonal bonds and self-reliance.

#### 3.3.1. Perceived Availability and Efficacy of Support

The most frequently cited reason for seeking help was confidence in the solution (33.5%), with youth expressing a strong belief that their confidants possess the experience and knowledge to provide meaningful guidance. Many described their parents or other trusted individuals as sources of wisdom and emotional stability, stating, *“My parents are the best, and they have more experience to be my mentors”*, and *“Because they have that brain to help and guide people to the right path.”* Similarly, perceived availability of support (23.2%) was a key motivator, with respondents emphasizing that they sought help from those who were consistently present and accessible, explaining, *“They are there for me when I am in need, and they advise me.”* Conversely, the absence of a trusted support system was a common deterrent, with some respondents stating, *“I don’t have the person who I can tell my secrets”*, or *“When I was young, I never had someone to talk to about my problem.”* These responses highlight the structural and interpersonal gaps that limit youth’s ability to access support, reinforcing patterns of social isolation.

#### 3.3.2. Trust, Safety, and Emotional Relief

Trust and safety (14.2%) played a crucial role in help-seeking behaviors, as youth emphasized the importance of confiding in those they trust to maintain confidentiality. Many expressed this sentiment explicitly, stating, *“Because they are the only people I trust, and I’m sure they can help me”*, and *“Because they are professional, and they will not disclose my problem to anyone.”* On the other hand, mistrust (11.8%) was also a significant deterrent, with some respondents expressing fear of betrayal: *“Because they are bad people that I cannot share with”*, and *“They tell their friends.”* The need for comfort and emotional relief (11.4%) was another key factor in help-seeking, as participants described how sharing their distress helped ease their burden: *“Problem shared is halfway solved”* and *“If I don’t tell anyone, I am the one who will suffer.”* In contrast, discomfort in disclosing emotions (1.4%) discouraged others from seeking help, with responses such as *“I don’t feel comfortable with sharing my problem”* and *“I am shy.”*

#### 3.3.3. Interpersonal Bonds vs. Self-Reliance

Many youth emphasized caring and understanding (13%) as motivators for seeking support, noting that they turn to those who are empathetic and nonjudgmental. This was reflected in statements such as *“I love sharing emotion with someone who will understand me”*, and *“Because they are good listeners, they understand and don’t judge.”* However, some avoided seeking help because they felt that others were indifferent to their struggles, stating, *“Many people won’t care about emotion”*, and *“No one tries to understand me, so I don’t try to talk.”* A strong theme of self-reliance (9.3%) emerged as a significant barrier to help-seeking, with many youth asserting their preference for independence over external assistance. Some framed this as personal strength, explaining, *“I like helping myself”*, and *“I trust myself.”* Others cited religious faith as their primary coping mechanism, stating, *“I don’t ask for help other than God”*, and *“I pray toward Mecca and read the Quran.”*

These findings illustrate a dual reality in Somali youth’s help-seeking behaviors: while many seek support from trusted individuals, others remain hesitant due to mistrust, emotional discomfort, or a deeply ingrained sense of self-reliance (see Table 5 for details).

### 3.4. Predictors of Perceived Support: Multiple Regression Analysis

A multiple regression analysis was conducted to examine the relationship between perceived support and key psychosocial and social capital predictors, including gender, age, emotional coping, help-seeking intention, religious coping, perceived community violence, scope of community, and trust in both bonding and bridging social capital. The overall model was statistically significant, F(9, 50) = 5.72, *p* < 0.001, accounting for 50.7% of the variance in perceived support (R^2^ = 0.507, adjusted R^2^ = 0.419), suggesting that these factors collectively play a substantial role in shaping perceived community support among displaced Somali youth. The standard error of the estimate (6.08) and Durbin-Watson statistic (DW = 2.23) indicated no serious autocorrelation, reinforcing the robustness of the model.

Among the predictor variables, the scope of community and trust in bonding social capital were significant predictors, suggesting that broader community definitions and stronger in-group trust enhance perceived support. Perceived community violence was also positively associated with perceived support, which could indicate that shared adversity may foster communal cohesion. Conversely, help-seeking intention was negatively associated with perceived support, possibly reflecting that those seeking formal help perceive less support from informal networks. Other variables, including emotional coping, religious coping, trust in bridging social capital, gender, and age, were not significant predictors. The non-significance of bridging trust may suggest that broader societal integration does not necessarily translate into greater perceived support. Similarly, religious coping did not predict perceived support, which may reinforce its role as an individual rather than a communal resilience factor (see Table 6).

## 4. Discussion

This study examined distress sources, coping strategies, and help-seeking behaviors among displaced Somali youth, highlighting the interplay of structural, familial, and psychological factors in shaping mental well-being. The findings emphasize the centrality of informal support systems, religious coping, and self-regulation while revealing significant barriers to formal mental health access. Regression analysis further underscores the role of social capital, particularly trust in bonding networks, in shaping perceived community support.

### 4.1. Sources of Distress and Implications for Psychosocial Well-Being

While a notable proportion of participants (i.e., 28.0%) reported no significant distress, most identified socio-economic hardship and displacement-related stressors as primary concerns. Economic instability and educational challenges were prominent, reinforcing prior research linking financial precarity to heightened mental health risks among refugee youth [1]. Beyond economic stress, fears of violence, police harassment, and systemic insecurity were prevalent, reflecting the precarious legal and social standing of urban refugees. These findings align with research on the chronic stress experienced by displaced youth navigating restrictive legal environments and systemic marginalization [54]. Family separation also emerged as a profound stressor, not only due to the emotional toll of absent familial support but also because of its broader implications within the collectivist Somali cultural framework (e.g., often forcing youth into caregiving or economic roles that heighten stress and strain resilience) [55,56]. In displacement, the absence of elders and extended kin fractures traditional coping structures, exacerbating distress, particularly when alternative support systems are weak [57].

Despite low explicit reporting of mental health concerns, qualitative responses reveal deeper psychological burdens, consistent with cultural stigmas that discourage direct acknowledgment of distress. Emotional suffering in collectivist societies is often framed through social or spiritual lenses rather than psychological terms, leading to somatization or avoidance-based coping [58,59]. Transnational anxieties—rooted in Somalia’s instability and the suffering of the diaspora—also highlight the enduring psychological toll of homeland crises. Displaced youth often experience “long-distance nationalism” and a sense of moral obligation toward their homeland, amplifying distress beyond immediate resettlement challenges [60,61]. Mental health interventions must acknowledge these dual realities, addressing both local stressors and the psychological weight of transnational ties.

### 4.2. Coping Strategies: The Centrality of Faith, Self-Regulation, and Social Networks

The findings reaffirm the centrality of religious coping among Somali refugee youth, consistent with literature highlighting faith as both an individual and collective resilience mechanism in displacement [15,18,22,39]. While spirituality provides psychological reassurance and social cohesion, it may not replace the broader protective role of social capital, particularly in contexts of systemic adversity faced by refugees. Although religious coping was among the most frequently reported strategies in this study (59.5%), it was not a significant predictor of perceived social support. This may reflect both conceptual and statistical limitations. While faith offers internal resilience and meaning, it does not necessarily correspond to the relational dimensions captured by instruments such as the MSPSS. Furthermore, its high prevalence (shaped by Eastleigh’s religious context and the near-universal Muslim identity of Somali youth) may have reduced its statistical power due to limited variability across participants. Over-reliance on faith in the absence of strong social networks may heighten vulnerability to isolation [41]; however, for Somali youth, religious beliefs also serve to strengthen peer and community bonds. In our framework, social capital refers to trust and reciprocal ties within interpersonal networks, whereas the religious coping practices reported here (e.g., Quran recitation and prayer) appeared more individualized than socially embedded. Nonetheless, participation in religious communities may indirectly foster social capital over time [15,62,63], even if this dynamic was not captured in our findings.

Alongside religious coping, self-care strategies were widely used, aligning with research on adaptive coping among refugee youth. While practices such as rest and self-encouragement provide accessible regulation methods, reliance on avoidance-based coping raises concerns. Avoidance may offer temporary relief but can lead to emotional suppression and unresolved distress, limiting long-term psychological resilience [64]. In contrast, social coping—particularly through same-sex friendships—plays a crucial role in emotional support, reflecting collectivist cultural norms [65]. However, while these peer networks provide immediate comfort, they do not necessarily encourage help-seeking beyond familiar circles. The low engagement in prosocial behaviors suggests that while social ties offer emotional reassurance, they do not always translate into outward support-seeking, potentially reinforcing cycles of internalized distress. This divergence in coping strategies underscores the need to strengthen informal support systems while integrating culturally congruent mental health interventions that promote both emotional processing and proactive engagement with broader community resources.

### 4.3. Help-Seeking Behavior and Social Capital: Trust in Informal Support and Barriers to Institutional Engagement

Consistent with coping patterns, help-seeking behaviors are predominantly oriented toward informal networks, with family and friends as primary sources of support. Mothers played a central role, reinforcing the importance of familial interdependence in Somali cultural contexts. Despite the prevalence of religious coping, few participants reported seeking direct emotional support from religious figures, which may indicate that spirituality serves as an internal resilience mechanism rather than an externalized support structure. Formal mental health services were rarely accessed, aligning with existing research on barriers to institutional engagement among refugee youth, including stigma, distrust, and perceived cultural misalignment of Western mental health models [23,66,67]. These findings point to the potential value of mental health initiatives embedded within culturally familiar structures, such as community-led support programs and peer-driven interventions, rather than relying solely on formal service provision.

This study also underscores the pivotal role of social capital in shaping perceived support. Trust in bonding networks was positively associated with perceived support, which may reflect the protective function of strong in-group cohesion. This aligns with research on social capital among refugee populations, emphasizing the buffering effects of dense community networks against stress and social exclusion [27,32,68]. Similarly, broader community engagement—measured by the scope of social ties—was positively associated with perceived support, suggesting that youth who define their communities more expansively may perceive greater access to social resources. Trust in bridging social capital, however, was not a significant predictor, implying that integration into broader societal networks may not necessarily translate into increased perceived support. This finding highlights the complexities of social capital, where in-group cohesion may be more immediately beneficial than cross-group connections, particularly in contexts of systemic discrimination.

Another notable finding was the positive association between perceived community violence and perceived support, suggesting that shared adversity may foster communal solidarity. This aligns with studies demonstrating that collective hardship can strengthen mutual aid within displaced communities [69]. However, such cohesion may be reactive rather than a stable resilience mechanism, emerging in response to crisis rather than serving as a sustained protective factor. Conversely, help-seeking intention was negatively associated with perceived support, possibly reflecting that individuals turn to formal assistance when informal support networks are weaker or unavailable. This aligns with theoretical perspectives that conceptualize help-seeking as a dynamic, non-linear process in which individuals navigate multiple decision points, each influencing whether they proceed with seeking help, the type of support accessed, and their level of engagement [70,71]. In contexts where strong community-based support structures exist, individuals may rely on these informal networks rather than institutional mental health services, reinforcing the complex interplay between social capital and formal help-seeking behaviors. It may reinforce the notion that informal help or bonding social capital can function as an alternative to professional interventions, particularly in communities where formal services are limited or culturally misaligned [72,73]. Collectively, these findings offer insight into the structural and relational conditions that shape perceived support among displaced refugee youth. By examining how different forms of social capital function within community life, the analysis foregrounds the importance of collective dynamics in shaping both social support and coping strategies. As such, the contribution of this study lies not in prescribing individual interventions but in illuminating the systemic conditions under which support is accessed, interpreted, and sustained.

### 4.4. Limitations

This study has several limitations that should inform the interpretation of its findings. The sample is restricted to Somali youth in urban displacement settings, and Eastleigh in specific, constraining generalizability to those in formal resettlement programs or other diaspora communities in high-income countries where sociopolitical contexts and access to resources differ. The cross-sectional design precludes causal inferences, limiting the ability to assess how distress, coping strategies, and perceived support evolve over time. As such, the findings should be interpreted as associative rather than explanatory. They suggest patterns of relationship among key variables rather than deterministic or causal effects. In addition, self-reported data introduce potential biases, particularly social desirability bias, as mental health stigma and cultural norms surrounding emotional expression may have influenced responses, leading to underreporting of psychological distress or avoidance-based coping. Additionally, a substantial proportion of participants did not report personal information (e.g., gender non-reporting at 28.0%), raising important methodological and interpretive concerns. Given the complex sociopolitical dynamics surrounding refugee identification and documentation, the relatively high percentage of missing data may reflect participants’ discomfort or reluctance to disclose demographic information.

While the mixed-methods approach enhances the study’s depth, the qualitative data were derived from brief open-ended responses rather than in-depth interviews, limiting the exploration of nuanced experiences and meaning-making processes. Furthermore, while social capital emerged as a critical factor in shaping perceived support, this study did not examine the dynamic interplay between bonding and bridging capital, nor how these forms of capital evolve or fluctuate in response to shifting sociopolitical conditions and displacement-related stressors. Additionally, structural barriers to formal mental health services—such as accessibility, affordability, and provider cultural competency—were not explicitly examined, despite their likely role in shaping help-seeking behaviors. Future research should incorporate longitudinal approaches to assess change over time, comparative studies across displacement contexts, and more in-depth qualitative methods to capture the complexity of coping and support systems within broader systemic and policy frameworks. Expanding the methodological design to include ethnographic fieldwork, focus groups, or participatory action research (PAR) could offer more nuanced insights into how individuals navigate stress, access informal support, and engage with broader community dynamics. These approaches are particularly suited to capturing the cultural, relational, and narrative dimensions of psychosocial experience in refugee contexts. Standard self-reported measures may fail to capture distress accurately in populations where emotional expression is constrained by cultural norms or prior stigma. Therefore, incorporating narrative-based methods, visual storytelling, or culturally adapted assessments can enhance understanding and engagement, ensuring that the depth of psychosocial distress is more effectively captured and addressed.

### 4.5. Implications for Practice and Policy

The findings highlight the critical need for community-driven mental health interventions that harness existing social capital while addressing barriers to formal care. Given the strong reliance on informal support networks, interventions should prioritize task-sharing and peer-based models that integrate trusted community figures, such as mothers and close friends, into structured psychosocial programs [74,75,76,77]. By training community peers to provide basic mental health support and facilitate connections to professional care, these approaches can expand service accessibility while maintaining cultural relevance. Such models not only mitigate the reluctance to engage with institutional mental health services but also foster collective coping and emotional expression in culturally congruent ways, reinforcing community resilience and sustainability [78,79,80]. This approach aligns with the UK DFID’s Sustainable Livelihoods Framework [81], which situates mental health support within broader strategies to strengthen community structures, highlighting social capital as a vital asset for resilience. Additionally, religious coping, while primarily an individualized strategy, remains an essential aspect of resilience for Somali refugee youth. Faith-based mental health initiatives, such as mosque-led psychoeducation and culturally adapted mindfulness practices, could enhance mental health literacy while maintaining cultural relevance [21,22,39].

The positive association between perceived community violence and social support suggests that communal cohesion strengthens in response to adversity. While this resilience is valuable, it also underscores the need for targeted interventions that provide proactive, rather than crisis-driven, social support mechanisms. Community-based psychosocial programs should focus on fostering trust and engagement in non-crisis contexts, ensuring that support networks remain strong even in the absence of acute threats [8,82,83]. Furthermore, the negative association between help-seeking intention and perceived support suggests that youth with weaker social networks may turn to formal services out of necessity; however, this does not imply access to adequate care. Instead, the lack of available and culturally responsive mental health services likely forces many to navigate distress without professional support, relying on informal coping mechanisms even when these are insufficient. This highlights both the structural gaps in mental health provisions and the critical role of community-based networks in bridging these service voids [65,84]. This finding reinforces the importance of bridging informal and formal care systems to prevent those with limited social resources from falling through service gaps.

Policy efforts must prioritize the development of culturally responsive mental health services that are embedded within existing social structures rather than relying on Western clinical models that may not align with Somali youth’s lived experiences. This includes expanding the availability of Somali-speaking mental health providers, integrating culturally adapted trauma-informed care, and embedding services within trusted community settings such as schools, religious institutions, and local organizations [7,85,86]. Given the limited availability of formal mental health services, equipping lay health workers, community leaders, and religious figures with basic psychosocial training can enhance both accessibility and cultural congruence, fostering a decentralized, community-based support system [78]. Such models not only mitigate reliance on under-resourced formal care but also strengthen social capital by leveraging existing networks of trust. Addressing structural barriers—including affordability, accessibility, and provider cultural competency—remains essential to ensuring that mental health interventions are both effective and sustainable in urban refugee contexts.

## 5. Conclusions

This study underscores the multifaceted nature of distress, coping, and help-seeking behaviors among displaced Somali youth, emphasizing the pivotal role of social capital, religious coping, and informal support networks in shaping their psychosocial well-being. While resilience is evident, particularly through faith-based and community-driven coping mechanisms, the heavy reliance on avoidance strategies and informal peer support highlights potential vulnerabilities in long-term mental health outcomes. The limited engagement with formal mental health services reflects broader structural and cultural barriers, reinforcing the necessity for interventions that are not only trauma-informed but also deeply embedded within culturally congruent frameworks of care. Given the centrality of bonding trust and communal support, mental health initiatives should move beyond individual-level interventions to leverage existing social structures, enhance community-based psychosocial programming, and bridge gaps between informal and formal support systems. Addressing these challenges requires a paradigm shift towards integrated, community-driven mental health strategies that respect cultural norms while fostering sustainable pathways to care, resilience, and well-being in displaced youth populations.

## Figures and Tables

**Table 1:** Characteristics of participants (N = 189).

	N	%
Gender		
Male	55	29.1%
Female	81	42.9%
Total Response	136	72.0%
Emotional Coping		
No	104	55.0%
Yes	67	35.4%
Total Response	171	90.5%
Religious Coping		
No	89	47.1%
Yes	83	43.9%
Total Response	172	91.0%
Help-Seeking (any problem)		
No	26	13.8%
Yes	145	76.7%
Total Response	171	90.5%
Help-Seeking (emotional problem)		
No	93	49.2%
Yes	77	40.7%
Total Response	170	89.9%
Perceived Scope of Community		
Family and friends only	36	19.0%
Same clan only	11	5.8%
All Somali people	67	35.4%
Neighbors including other cultural/ethnic groups	42	22.2%
Total Response	156	82.5%
	M	SD
Age (15–25)	19.88	2.215

**Table 2:** Emotional distress.

Theme	Quote	N	%
None		53	28.0%
Basic needs, livelihood, education	Job, I am worried about job, am jobless and it’s the only worry I have. I worry about my fee for my IT classes My exams. Unemployment	30	15.9%
Refugee-related adversities	I am scared of the people who once tried to kill my father and harm my family to come back. The police harassing youths in the community. Police harassment and violence happening in my community. How to leave Kenya.	25	13.2%
Family-related issues	I am very worried about my family because I am not with them, especially my mum. I worry about my children who are not with me/far away. My sick children	32	16.9%
Mental health	I have nightmares. I think a lot. The life I am in is making me worry so much. Always I think about how to forget and make it on the way. Forgetting and thinking a lot. Some sickness in my head also punishment from the school.	14	7.4%
Community and Somalia	My most worrying thing is my community and how I will help them. The Somali youth who are suffering all over the world and natural disasters. I most worried because of people of Eastleigh, I want to help their emotional suffering. Worried about the election. Still the country [Somalia] has no peace. Problems in Somalia and the insecurity in Kenya. The conflict between the Somali community.	10	5.3%
Not reported		25	13.2%
Total (valid)		164	86.8%

**Table 3:** Types of coping (multiple responses).

Category	Example	Frequency (n)	% Of Total Cases (189)	% Of Total Responses (505)
Religion	Religious Coping	113	59.5%	22.4%
	Reading/Listening to Quran Praying Visit to Mosque Worship Repent my sins			
Self-care and Self-regulation		112	60.5%	22.2%
	Physical Self-Care	57	30%	11.3%
	Sleep Rest Bath Drink Water			
	Psychological Self-Care	44	23.7%	8.8%
	Self-Encouraging Seek Happiness Reading Diary and Achievement Self-talk Reading books about depression Minimize my thoughts			
	Advanced Self-Care	11	6.8%	2.2%
	Breathing Meditation			
Leisure		35	18.4%	6.9%
	Physical Activities	16	8.4%	3.2%
	Exercise Play football/Soccer Walk Running Swim			
	Passive Leisure	19	10%	3.7%
	Listening to something that makes me happy (i.e., music) Reading books Watching movie/sport Play/TV			
Social		49	25.8%	9.7%
	Socializing	43	22.6%	8.5%
	See someone I love (include family, friends) Mix with community Going out with friends Party Try not to be alone			
	Prosocial Activity	6	3.2%	1.2%
	Help others Do good things			
Help-seeking		103	56.4%	20.4%
	Formal Help-Seeking	23	12.1%	4.5%
	Going to school Counselling Seeking advice Rehab Support group			
	Informal Help-Seeking	10	5.3%	2%
	Looking for a good person Getting encouragement from the community			
	Talk to Others	70	39%	13.9%
	Talk to someone (family, friend, people) Sharing Ask advice			
Problem solving		8	4.2%	1.6%
	Change environment Finding the cause			
Emotional burst		10	5.3%	2%
	Cry Scream			
Avoidance		30	15.8%	6%
	Distracting	16	8.4%	3.2%
	Make myself busy Move myself from the stress Work			
	Avoidance	14	7.4%	2.8%
	Try to forget Minimize the cause Avoid thinking about the problems			
Being alone		11	5.8%	2.2%
	Going away from people Try to find quiet place Being alone in a dark room Finding quiet places			
Others		34	8.9%	6.7%
	Showing patience Doing medication Curse the devil Pretend to be happy Leaving Africa Being humorous Think a lot			
Total		505		

**Table 4:** Whom to share problems with.

Category	Group	Frequency (n)	% Out of Case # (190)	% Total Responses (206)
**Informal**				
	Family Members			
	a. Mother	17	8.9%	8.3%
	b. Parents	15	7.9%	7.3%
	c. Spouse	1	0.5%	0.5%
	d. Sibling	5	2.6%	2.4%
	e. Family Members/Relatives	49	25.8%	23.8%
	Friends			
	a. Same Sex Friend	71	37.4%	34.5%
	Community members			
	a. Neighbors	5	2.6%	2.4%
	b. Someone Else (Unspecified)	24	12.6%	11.7%
	Religious figure			
	a. God (Allah)	2	1.1%	1%
**Formal**				
	Teacher	9	4.7%	4.4%
	Professional	6	3.2%	2.9%
	Other (lectures)	2	1.1%	1%
**Total**		**206**		

**Table 5:** Reasons for seeking help and for not seeking help (n = 211).

Themes	Reason for Help-Seeking	Frequency (%)	Reason for Not Asking for Help	Frequency (%)	Total Frequency (%)
Available Support	“Maybe they can help me with my problems and my emotional suffering.” “My mother. Because she always gives courage.” “Because I think they will help me solve my problems or half of them.” “They are there for me when I am in need, and they advise me.” “Because they are close to me, and I always take them as my friends.”	49 (23.44%)	“I don’t have the person who I can tell my secrets.” “I tried many times, but I did not succeed.” “Because when I was young, I never had someone to talk to about my problem.”	4 (11.76%)	53 (21.81%)
Confidence in solution	“Because they create awareness among us.” “Because they can assist you any causes.” “My parents are the best and they have more experience to be my mentors.” “People who have knowledge on how to overcome depression.” “Because they have that brain to help and guide people to the right path and help them solve their problems.” “To feel relieved and they will surely help me get rid of the problem.”	67 (32.06%)	“I don’t talk to them about my problem because they are suffering too.” “I listen to lectures which is helpful.”	2 (5.88%)	69 (28.40%)
Trust and Safety (confidentiality)	“Because they are the only people I trust and I’m sure they can help me.” “Because they are my people.” “Because that person may be the one, I trust.” “I feel safe every time I share my problems with them.” “Because they are professional, and they will not disclose my problem to anyone.”	30 (14.35%)	“Because they are bad people that I cannot share with.” “Because I don’t trust anyone.” “They tell their friends.”	4 (11.76%)	34 (13.99%)
Comfort and Relief	“Because they are good listeners.” They understand and don’t judge.” “Because they help me to forget it and do anything to make me happy (avoidance).” “Because I feel relieved when I talk about it [emotional distress].” “They calm me down.” “So that I get relief.” “It helps me reduce my suffering and stress. Problem shared is halfway solved”. “Because if I don’t tell anyone I am the one who will suffer.”	24 (11.48%)	“Because I don’t feel free with them.” “I don’t feel comfortable with sharing my problem.” “I am shy.”	3 (8.82%)	27 (11.11%)
Caring and Understanding	“I love sharing emotion with someone who will understand me.” “Because somehow they do understand me.” “Because they know my problem.” “Because they care about me.” “Because they are understanding and caring.” “Because they are good listeners, they understand and don’t judge.” “Because they are the only ones who can understand my feelings.”	26 (12.44%)	“Because many people won’t care about emotion.” “No one tries to understand me, so I don’t try to talk.”	2 (5.88%)	28 (11.52%)
Interdependence vs. Self-dependence	“The only person I share my emotional suffering is my mom. She has the right for everything about me.” “Because they are my people/my family.” “Because we all are human being.” “Because the Somali people help each other.” “Because it is a human being.”	9 (4.31%)	“I like helping myself.” “I don’t like asking others for help.” “Because I don’t ask help other than God.” “Problem I have I don’t tell people.” “I don’t like asking others for help.” “I like helping myself.” “I trust myself.” “I don’t like asking others.” “I pray God, rather than talk to others.” “I seek help from God.” “Because I pray toward Mecca and read holy Quran.”	20 (58.8%)	29 (11.93%)
Others		4 (1.91%)		-	4 (1.65%)
		209		34	243

**Table 6:** Multiple regression model predicting perceived support.

Predictors	B	SE	β	t	*p*	95% CI (Lower)	95% CI (Upper)
(Constant)	−3.702	9.622		−0.385	0.702	−23.028	15.624
Gender	−2.267	1.852	−0.138	−1.224	0.227	−5.987	1.453
Age	0.532	0.373	0.149	1.427	0.16	−0.217	1.282
Emotional Coping	1.751	1.863	0.109	0.94	0.352	−1.991	5.493
Religious Coping	1.04	1.77	0.066	0.588	0.559	−2.515	4.594
Help-Seeking (intention)	−3.278	1.628	−0.206	−2.014	**0.049**	−6.548	−0.008
Scope of Community	4.404	1.29	0.417	3.415	**0.001**	1.814	6.995
Perceived community violence	2.503	0.812	0.346	3.084	**0.003**	0.873	4.133
Trust in bridging SC	−1.811	1.333	−0.175	−1.359	0.18	−4.488	0.866
Trust in bonding SC	3.901	1.504	0.343	2.594	**0.012**	0.88	6.922

## Data Availability

Due to confidentiality and ethical considerations, the data supporting the findings of this study are not publicly available. Access to data is restricted to protect participant privacy and comply with ethical guidelines approved by the Institutional Review Board and Community Ethics Advisory Board.
